# Antimicrobial Activity of Some Essential Oils against Methicillin-Susceptible and Methicillin-Resistant *Staphylococcus pseudintermedius*-Associated Pyoderma in Dogs

**DOI:** 10.3390/ani10101782

**Published:** 2020-10-01

**Authors:** Francesca Paola Nocera, Simone Mancini, Basma Najar, Fabrizio Bertelloni, Luisa Pistelli, Anna De Filippis, Filomena Fiorito, Luisa De Martino, Filippo Fratini

**Affiliations:** 1 Department of Veterinary Medicine and Animal Production, Via Delpino 1, University of Naples “Federico II”, 80137 Naples, Italy; francescapaola.nocera@unina.it (F.P.N.); anna.defilippis@unina.it (A.D.F.); filomena.fiorito@unina.it (F.F.); 2 Department of Veterinary Sciences, Viale delle Piagge 2, University of Pisa, 56124 Pisa, Italy; simone.mancini@unipi.it (S.M.); fabrizio.bertelloni@unipi.it (F.B.); filippo.fratini@unipi.it (F.F.); 3 Department of Pharmacy, Via Bonanno 6, University of Pisa, 56126 Pisa, Italy; basmanajar@hotmail.fr (B.N.); luisa.pistelli@unipi.it (L.P.)

**Keywords:** *Staphylococcus pseudintermedius*, canine skin disorder, essential oils, MIC, MBC

## Abstract

**Simple Summary:**

Pyoderma is one of the most common diseases in dogs, and *Staphylococcus pseudintermedius*, a Gram-positive coagulase-positive bacterium, represents the most common infectious agent causing canine pyoderma. Since multidrug-resistant *S. pseudintermedius* strains have become a relevant threat in veterinary medicine, this study aimed to test the antimicrobial properties of some essential oils (EOs) against *S. pseudintermedius* strains isolated from dogs suffering from pyoderma. The obtained findings demonstrated a clear *in vitro* efficacy of some tested EOs against clinical methicillin-resistant and methicillin-sensible *S. pseudintermedius* strains. The applicability and efficacy of EOs in cases of canine pyoderma supported by *S. pseudintermedius* could be beneficial for both dogs and pet owners, who are inevitably exposed to this zoonotic bacterium.

**Abstract:**

This study aimed to test *in vitro* the antimicrobial activity of 11 essential oils (EOs) against four methicillin-resistant *Staphylococcus pseudintermedius* (MRSP) and four methicillin-susceptible *S. pseudintermedius* (MSSP) clinical isolates. The obtained findings demonstrated a clear *in vitro* efficacy of some tested EOs against both MRSP and MSSP strains. Particularly, modal minimum inhibitory concentration (MIC) values ranging from 1:2048 *v*/*v* for *Melissa officinalis* against an MSSP strain to 1:256 *v*/*v* for *Cymbopogon* *citratus* against all MRSP strains were observed. The best results, highlighting a modal MIC value of 1:1024 *v*/*v* for all tested isolates, was provided by *Cinnamomum zeylanicum*. Intriguingly, *Cinnamomum zeylanicum* showed, in many cases, a correspondence between minimum bactericidal concentration (MBC) and MIC values, indicating that the inhibiting dose is also often bactericidal. Moreover, a mild antibacterial and bactericidal activity against both MRSP and MSSP isolates was detected for the other tested EOs. Considering the zoonotic potential of *S. pseudintermedius* and the increased dissemination of multidrug-resistant strains, the employment of EOs could be useful for the treatment of canine pyoderma. Since antibiotic resistance has become the most urgent issue, from the perspective of the One Health initiative, alternative therapeutic approaches are desirable to limit the use of antibiotics or to improve the efficacy of conventional therapies.

## 1. Introduction

In recent years, alternative treatments, including essential oils (EOs), have become very popular as natural remedies in human and veterinary medicine. The establishment of new approaches to conventional therapies, using selected EOs, for the treatment of canine skin disorders was the objective of this study.

Skin disorders are very common in pet animals, and the most frequent causes are allergies from parasites such as fleas, environmental allergies, and adverse food reactions. However, all alterations of the skin surface microenvironment promote bacterial multiplication [1]. It is known that *Staphylococcus pseudintermedius* is the staphylococcal species most frequently isolated from dogs suffering from pyoderma. This coagulase-positive bacterium is an opportunistic canine skin pathogen that inhabits healthy dogs, and its nasal carriage was also demonstrated in healthy pet-owning household members [2].

In the past, *S. pseudintermedius* isolates were generally susceptible to β-lactam antibiotics; however, since over a decade, methicillin-resistant strains (MRSP; methicillin-resistant *S. pseudintermedius*) have emerged as a significant health problem in pet animals. Over the years, MRSP has been reported with increasing frequency [3,4,5]. Furthermore, MRSP strains often show multidrug resistance profiles worldwide, including resistance to several classes of antimicrobial drugs [6].

In recent years, several studies were carried out both *in vivo* and *in vitro* on the efficacy of some EOs against the etiological agents of pyoderma in dogs [7,8,9]. Many EOs can be used in these skin disorders; however, thanks to their bioactive chemical compounds, some of them are effective tools especially against Gram-positive bacteria [10]. In particular, several EOs derived from plants belonging to the *Lamiaceae* family have shown a significant antibacterial activity [11]. Moreover, EOs characterized by high percentages of thymol and carvacrol show a remarkable membrane-damaging activity in bacteria. In this work, the EOs of savory, lemon balm, and basil were selected as representatives of this important family of medicinal plants. The other EOs selected for this research were obtained from plants whose antibacterial activity has been less studied than that of the botanical species belonging to the *Labiatae* family.

With regard to the antibacterial activity of manuka essential oil, not many data are available; however, some recent researches reported good activity against *Staphylococcus* spp. and in general against Gram-positive bacteria, thanks to the presence of some compounds such as leptospermone and isoleptospermone [8,12]. In particular, one study analyzed the efficacy of manuka EO against *S. pseudintermedius* isolated from canine pyoderma and otitis samples, highlighting its excellent activity against all these bacterial isolates [13].

Few scientific works reported the antibacterial activity of some resins such as myrrh, although many important biological activities are traditionally recognized [14,15]. Cinnamon EO is effective against many Gram-positive and Gram-negative bacteria and it is also used in the food industry with considerable results [16]. The antibacterial activity of eucalyptus and lemongrass EOs was reported in numerous studies available in the literature [17,18]. On the other hand, less experimental evidence is available to demonstrate the antibacterial efficacy of verbena EO [19,20]. Recent studies supported the antibacterial effectiveness of the EOs obtained from many citrus fruits, including *Citrus aurantium*, even though they did not show a particularly high activity [21,22].

The antibacterial activity of *Cannabis sativa* EO is one of the aspects considered most recently, since other biological activities of this plant have received more attention from the scientific world. A recent study conducted in Italy showed how the presence of some compounds, such as α- and β-pinene, β-myrcene, and β-caryophyllene, promote the antibacterial activities of essential oils derived from *Cannabis sativa* against different microorganisms [23].

The topical application of EOs could be a promising alternative therapeutic tool in dog skin disorders, such as pyoderma. For this reason, the main purpose of this research was to evaluate the inhibitory and bactericidal activity of different commercially available EOs potentially viable in therapy against methicillin-susceptible and methicillin-resistant *S. pseudintermedius* isolates from canine pyoderma.

## 2. Materials and Methods

### 2.1. Essential Oils

The EOs of *Citrus aurantium* L. (Ca), *Ocimum basilicum* L. (Ob), *Eucalyptus globulus* Labill. (Eg), *Aloysia triphylla* (L’Hér.) Britton (At), *Cymbopogon citratus* (DC.) Stapf (Cc), *Melissa officinalis* L. (Mo), *Cinnamomum zeylanicum* Blume (Cz), *Commiphora molmol* (Engl) Engl. ex Tschirch (Cm), *Satureja montana* L. (Sm), and *Leptospermum scoparium* J.R. Forst and G. Forst (Ls) were purchased directly from the market (FLORA^®^, Pisa, Italy); *Cannabis sativa* L. (Cs) EO was purchased from the company GADOI^®^ (Badia Calavena, Verona, Italy). According to the indications on the label, EOs were obtained by steam distillation, except for the *Citrus aurantium* L. EO, which was obtained by cold pressing.

### 2.2. Chemical Composition of the Tested EOs

A chemical characterization of the EOs was carried out by GC-EIMS (Gas chromatography coupled with electron impact mass spectrometry) at the Department of Pharmacy, University of Pisa (Pisa, Italy). Each EO was diluted to 5% in HPLC-grade *n*-hexane and then injected into a GC-EIMS apparatus. GC-EIMS analyses were performed with an Agilent 7890B gas chromatograph (Agilent Technologies Inc., Santa Clara, CA, USA) equipped with an Agilent HP-5MS (Agilent Technologies Inc., Santa Clara, CA, USA) capillary column (30 m × 0.25 mm; coating thickness 0.25 μm) and an Agilent 5977B single-quadrupole mass detector (Agilent Technologies Inc., Santa Clara, CA, USA). Analytical conditions were as follows: injector and transfer line temperatures of 220 °C and 240 °C, respectively; oven temperature programmed from 60 °C to 240 °C at 3 °C/min; carrier gas helium at 1 mL/min; injection of 1 μL; split ratio 1:25. The acquisition parameters were the following: full scan; scan range: 30–300 *m*/*z*; scan time: 1.0 s. Identification of the constituents was based on a comparison of the retention times with those of the authentic samples, comparing their linear retention indices relative to a series of *n*-hydrocarbons. Computer matching was also used against commercial (NIST 14 and ADAMS) and laboratory-developed mass spectral libraries built up from pure substances and components of known oils and MS literature data [24,25,26,27,28,29]. EOs were stored at 4 ± 2 °C in the dark until their use.

### 2.3. Phenotypic and Genotypic Identification of Bacterial Isolates

Eight veterinary clinical isolates, named from 1 to 8, using four MRSP (methicillin-resistant *S. pseudintermedius*) and four MSSP (methicillin-sensible *S. pseudintermedius*) strains, were selected from the bacterial stocks stored at −80 °C in Microbank™ vials (Pro-lab Diagnostics, Richmond Hill, ON, Canada) belonging to Microbiology Laboratory of the Department of Veterinary Medicine and Animal Production of the University of Naples Federico II (Naples, Italy). Briefly, from dogs, attending the Veterinary University Teaching Hospital of Naples, skin samples were collected to perform bacteriological analysis and antimicrobial susceptibility tests. Upon arrival at the laboratory, specimens were cultured on Columbia Nalidixic Acid agar (CNA) with 5% sheep blood and on mannitol salt agar (MSA) plates (Oxoid, Milan, Italy) and incubated aerobically at 37 °C for 24 h. *Staphylococcus* spp. presumptive colonies were subjected to a first identification using standard techniques: colony morphology, Gram staining, and coagulase and catalase tests. Then, all the isolates were identified by matrix-assisted laser desorption ionization time-of-flight mass spectrometry (MALDI-TOF-MS) (Bruker Daltonik, Germany) using fresh colonies grown on Columbia CNA agar. Specifically, the bacterial colony was first inoculated in the plate for mass spectrometry and, then, 1 µL of the organic matrix, cinnamic acid, was added to the sample. Afterward, the plate was placed in the equipment for MALDI-TOF-MS analysis. The identification was based on the score value released by the manufacturer’s instructions. Values from 2.3 to 1.9 indicated the best identification of genus and species [30].

For the molecular characterization of the stored strains, each *S. pseudintermedius* isolate was cultured again on MSA plates with incubation at 37 °C overnight. The bacterial DNA extraction of the isolates was carried out by using the commercial Isolate II Genomic DNA kit (Bioline, London, UK) and following the manufacturer’s instructions. The obtained bacterial DNA was stored at −20 °C.

All isolates were tested by polymerase chain reaction (PCR) for the species-specific *nuc* and *hlb* genes (Table 1) to further confirm the proteomic identification by MALDI-TOF-MS. *S. pseudintermedius* ATCC^®^ 49444TM was used as positive control. Indeed, to distinguish the species belonging to the *Staphylococcus intermedius* group (SIG), a species-specific multiplex PCR as a function of the thermo nuclease (*nuc*) gene was generally performed [31].

*S. pseudintermedius* constitutively produces β-hemolysin. On the basis of the *S. pseudintermedius* ED99 complete genome, deposited in Genbank, a new pair of primers for *hlb* gene, which enable the analysis of *S. pseudintermedius* strains, were designed [32]. These investigations allow better identifying *S. pseudintermedius* and distinguishing it from the other members of the SIG group.

### 2.4. Genotypic and Phenotypic Antibiotic Resistance of Isolates

The bacterial DNA was also tested for the presence of the *mec*A gene [33] (Table 1), which was validated using in-house positive and negative control strains, for which both phenotypic and genomic data were available. The veterinary clinical MRSP and MSSP isolates were further analyzed using the Kirby-Bauer disc diffusion susceptibility method for the occurrence of antibiotic resistance profiles. All isolates were assessed for their susceptibility to the following panel of antimicrobials: amoxicillin-clavulanate (AMC, 20/10 μg), ampicillin (AMP, 10 μg), ceftriaxone (CRO, 30 μg), clindamycin (CD, 2 μg), ciprofloxacin (CIP, 5 μg), erythromycin (E, 15 μg), enrofloxacin (ENR, 5 μg), gentamicin (CN, 10 μg), imipenem (IMI, 10 μg), linezolid (LNZ, 30 μg), oxacillin (OX, 1 μg), penicillin (P, 10 IU), streptomycin (S, 10 μg), sulfamethoxazole-trimethoprim (SXT, 1.25/23.75 μg), tetracycline (TE, 30 μg), tobramycin (TOB, 10 μg), and vancomycin (VA, 30 μg). The isolated strains were classified as susceptible (S) or resistant (R) according to the Clinical and Laboratory Standards Institute [34] and European Committee on Antimicrobial Susceptibility Testing [35] guidelines. For streptomycin and vancomycin breakpoints, those recommended by the French Society for Microbiology (http://www.sfm-microbiologie.fr) were employed.

### 2.5. Minimum Inhibitory Concentration (MIC) and Minimal Bactericidal Concentration (MBC) Determinations

Minimal inhibitory concentration (MIC) was determined using a twofold serial microdilution method, as previously described [36], at the Department of Veterinary Sciences, University of Pisa (Pisa, Italy). Ninety-five microliters of BHI (Brain Hearth Infusion, Thermo Fischer, Milan, Italy) broth was distributed in a 96-well microtiter plate; the EO dilution stock was prepared in BHI broth with dimethyl sulfoxide (DMSO) added to a final ratio of 1:3:4 (EO:DMSO:BHI, *v*/*v*/*v*). Ninety-five microliters of EO dilution was dispensed in the first well of each series, and then twofold dilutions were performed. Bacterial suspensions, adjusted to 0.5 on the McFarland standard turbidity scale (approximately 1.5 × 10^8^ colony-forming units (CFU)/mL), were added to each well to reach a final volume of 100 μL. Wells containing bacterial suspension and BHI or BHI alone were employed as positive and negative controls, respectively. Microplates were incubated at 37 °C for 24 h in a humid chamber. EO MIC determinations were performed in triplicate.

Minimal bactericidal concentration (MBC) was determined by streaking one drop from each well that showed a concentration of EO equal to or higher than the MIC value on TSA (Trypticase Soy Agar, Thermo Fischer Scientific, Milan, Italy). TSA plates were incubated at 37 °C for 24 h. MBC values were determined as the lowest concentrations that did not allow colonies growth.

## 3. Results

### 3.1. S. pseudintermedius Strain Identification

The eight isolated strains were identified, with a log (score) of ≥2.0, as *S. pseudintermedius* by MALDI-TOF-MS. Moreover, all isolates harbored the species-specific *nuc* and *hlb* genes, thus confirming the proteomic identification by MALDI-TOF-MS.

### 3.2. Antibiotic Resistance Patterns of the S. pseudintermedius Isolates

Four isolates were MRSP strains carrying the *mec*A gene. Interestingly, they also displayed multidrug-resistant profiles, showing resistance to at least three different antibiotic classes. In fact, MRSP antimicrobial susceptibility results (Table 2), obtained from Kirby-Bauer disc diffusion testing, showed a complete resistance to amoxicillin-clavulanate, ampicillin, ceftriaxone, ciprofloxacin, erythromycin and sulfamethoxazole-trimethoprim (100%). The MSSP isolates displayed broad resistance to ampicillin and penicillin (100%) but revealed broad susceptibility to the other tested antibiotics, as shown in Table 2. No resistance was observed to vancomycin and linezolid for both MRSP and MSSP isolates.

### 3.3. Essential Oil Composition

The percentage of identified compounds ranged between 87.6% of *Leptospermum scoparium* to 100% of *Citrus aurantium* (Table 3). Limonene was the main compound identified in *Citrus aurantium* with a percentage of 92.6% followed by 1,8-cineole (84.2%) in *Eucalyptus globulus* and by *trans*-cinnamaldehyde (63.2%) in *Cinnamomum zeylanicum*.

Other compounds characterized by considerable antibacterial activity found in good amount were the following: myrcene (16.1%) and β-caryophyllene (20.8%) in *Cannabis sativa* EO, and curzerene (17.5%) in *Commiphora molmol* EO. Other compounds found in significant quantities with documented antibacterial properties were carvacrol (45.4%) in *Satureja montana*, geranial (40.1%) and neral (32.6%) in *Cymbopogon citratus*. Significant amounts of these antibacterial compounds were also found in *Melissa officinalis* EO with 36.5% and 29% for geranial and neral, respectively. A high percentage of limonene (31.1%) was also evidenced in *Aloysia triphylla* EO. Furanoeudesma-1,3-diene, a particular compound with antimicrobial activity [37], was also present in high percentage in *Commiphora molmol* EO (33.7%). The compounds most represented in *Leptospermum scoparium* EO, which have demonstrated a remarkable antibacterial activity against several bacterial isolates, were *cis*-calamenene (22.7%) and leptospermone (19.2%), as already reported in a previous study [38].

### 3.4. Antibacterial Activity of the Tested Essential Oils

MIC and MBC values are reported in Table 4 and Table 5, respectively. Among the tested EOs the best results against all strains of *S. pseudintermedius* were provided by *Cinnamomum zeylanicum*, *Melissa officinalis*, *Leptospermum scoparium*, *Satureja montana*, and *Cymbopogon citratus* EOs with modal MIC values ranging from 1:2048 *v*/*v* for *Melissa officinalis* EO against an MSSP isolate (isolate number 8) to 1:256 *v*/*v* for *Cymbopogon citratus* EO against all MRSP isolates. The other tested EOs showed instead a mild antibacterial and bactericidal activity against both MRSP and MSSP isolates with MIC modal values ranging from 1:256 *v*/*v* for *Commiphora molmol* EO against all MRSP isolates to 1:16 *v*/*v* for *Cannabis sativa* EO against all MRSP isolates.

Specifically, *Cinnamomum zeylanicum* EO provided the best results, highlighting a modal MIC value of 1:1024 *v*/*v* for all tested isolates, both MRSP and MSSP. With regard to this EO, another important aspect is that, in many cases (5/8), the value of MBC also corresponded to the value of MIC, indicating that the inhibitory dose is often also bactericidal. *Melissa officinalis* EO showed similar antibacterial activity with MIC values of 1:1024 *v*/*v* with the exception of one MRSP isolate (number 2) for which the value was 1:512 *v*/*v* and one MSSP isolate (number 8) for which the value was 1:2048 *v*/*v*. The bactericidal activity was more effective against MSSP isolates (in three cases out of four, the value of MBC was 1:1024 *v*/*v*, isolate numbers 5, 7, and 8) compared to MRSP isolates for which the values of MBC were in all cases equal to 1:512 *v*/*v*.

In spite of the susceptibility of the isolates, *Leptospermum scoparium* EO showed good antibacterial and bactericidal activities with MIC values ranging from 1:512 *v*/*v* to 1:1024 *v*/*v* and MBC values ranging from 1:256 *v*/*v* to 1:512 *v*/*v*. Furthermore, in the case of *Satureja montana* EO, higher inhibitory activity was highlighted against MSSP isolates compared to MRSP with MIC values ranging from 1:512 *v*/*v* to 1:1024 *v*/*v* for the former versus MIC values ranging from 1:256 *v*/*v* to 1:512 *v*/*v* for the latter. A similar trend was shown for bactericidal activity.

A remarkable difference in inhibitory activity between MRSP and MSSP isolates was found in *Cymbopogon citratus* EO. In this case, the MIC values for the MRSPs were 1:256 *v*/*v*, which can be interpreted as a modest inhibitory activity, while, for the MSSPs, the MIC values were 1:1024 *v*/*v*. However, it should be noted that, for the MRSP isolates, the values of MBC corresponded to the values of MIC.

## 4. Discussion

Canine bacterial skin infections represent the main reason behind presentation in small animal practice. *S. pseudintermedius*, a normal inhabitant of the skin and mucosa of dogs, is the major causative agent of superficial pyoderma [4]. The increasing spread of multidrug-resistant *S. pseudintermedius* strains has become a relevant challenge in veterinary medicine [4]. Repeated antibiotic treatments may then increase the risk of selecting for multidrug-resistant bacteria, one of the most relevant current threats to public health. The close contact between animals and their owners provides opportunities for bacterial transmission, including MRSP strains [39].

Studies on alternative nonantibiotic substances need to be explored in order to carry out new therapies for disease treatments. In the present paper, the obtained promising *in vitro* results demonstrated a clear efficacy of some EOs against canine MRSP and MSSP. Particularly, some tested EOs demonstrated a relevant antibacterial activity against all tested strains. Precisely, *Cinnamomum zeylanicum* EO provided the best results against both MRSP and MSSP, showing almost always a concordance in MBC and MIC values. This study finding confirms the efficacy of *Cinnamomum zeylanicum* EO, whose antibacterial activity was already reported against bacterial isolates from human orofacial infections [40] and against the food-borne pathogens *Staphylococcus aureus* and *Escherichia coli* [41]. Moreover, *in vivo* studies also reported the activity of *Cinnamomum zeylanicum* EO against both planktonic and biofilm forms of Gram-positive and Gram-negative bacteria [42].

Herein, *Melissa officinalis* EO showed similar antibacterial activity against both MRSP and MSSP, and a more effective bactericidal activity against MSSP isolates. *Melissa officinalis* EO properties are already known in veterinary medicine. Indeed, Ehsani et al. [43] reported the possible appropriate application of *Melissa officinalis* EO in the food industry, due to its antioxidant and antibacterial properties against four important food-borne bacteria (*Salmonella typhimurium*, *Escherichia coli*, *Listeria monocytogenes*, and *Staphylococcus aureus*). Furthermore, a strong antimicrobial activity of *Melissa officinalis* EO against bacterial microflora isolated from fish was also described [44]. However, in this study, we also obtained good results for *Leptospermum scoparium*, *Satureja montana*, and *Cymbopogon citratus* EOs against all selected *S. pseudintermedius* strains.

Since this preliminary investigation highlighted that some of the tested EOs proved to be valuable tools in pyoderma therapy, it seems desirable to continue to perform further studies on EOs, in order to assess their efficacy in not only *in vitro* but also *in vivo* trials. Particularly*, Cinnamomum zeylanicum, Melissa officinalis*, *Cymbopogon citratus*, and *Satureja montana* EOs may represent promising and valid candidates for *in vivo* use. Interestingly, the efficacy demonstrated by *Melissa officinalis* EO makes it the best prospect for *in vivo* use.

However, it is also necessary to remember that the yield in essential oil from this plant is extremely low, often below 0.1%; thus, for this essential oil, it would be absolutely desirable to use it in a mixture with other oils [45].

From some of the tested EOs, we could have expected a greater effectiveness in antibacterial action in view of the data reported in literature; however, the differences among the compounds are probably linked to their different biological activities [46]. Hence, mixtures of the EOs could also be considered to determine their potential synergistic action. The extremely low dosages needed for EOs allow minimizing any adverse effects, giving effective alternatives to topical treatment with antibiotics. It is worth noting that these nonantibiotic treatment strategies might help to reduce the severity of canine *S. pseudintermedius* infections and to limit further colonization, thereby also preserving the health of pet owners.

## 5. Conclusions

In our knowledge, the present study revealed for the first time the antimicrobial properties of our selected EOs against both MRSP and MSSP strains isolated from dogs suffering from pyoderma. In particular, *Cinnamomum zeylanicum* and *Melissa officinalis* showed the strongest antibacterial activity. Our results underline that EOs may be considered promising therapeutic agents to treat infections caused by zoonotic multidrug-resistant *S. pseudintermedius* strains, which are becoming more and more difficult to manage.

## Figures and Tables

**Table 1:** Primer sequences, amplicon sizes, and amplification programs. F, forward; R, reverse.

Gene	Primer Sequences	Amplicon Size (bp)	Amplification Program
*nuc*	F: TRGGCAGTAGGATTCGTTAA R: CTTTTGTGCTYCMTTTTGG	926	94 °C 5 min; 94 °C 30 s, 58 °C 60 s, 72 °C 90 s, for 30 cycles; 72 °C 5 min.
*hlb*	F: GACGAAAATCAAGCGGAA R: TCTAAATACTCTGGCGCAC	734	94 °C 2.5 min; 94 °C 30 s, 56 °C 30 s, 72 °C 1 min, for 30 cycles; 72 °C 10 min.
*mecA*	F: TCCACCCTCAAACAGGTGAA R: GGAACTTGTTGAGCAGAGGT	139	94 °C 5 min; 94 °C 30 s, 55 °C 40 s, 72 °C 30 s, for 30 cycles; 72 °C 5 min.

**Table 2:** Antimicrobial resistance patterns of methicillin-resistant and methicillin-susceptible *Staphylococcus pseudintermedius* isolated from canine pyoderma cases.

Antibiotics	Isolates							
	1	2	3	4	5	6	7	8
AMC	R	R	R	R	R	R	S	R
AMP	R	R	R	R	R	R	R	R
CRO	R	R	R	R	S	S	S	S
CD	S	R	R	R	S	S	S	S
CIP	R	R	R	R	S	S	S	S
E	R	R	R	R	S	S	R	S
ENR	R	R	R	S	S	S	S	S
CN	S	R	R	S	S	S	S	S
IMI	S	R	R	R	S	R	S	S
LNZ	S	S	S	S	S	S	S	S
OX	R	R	R	R	S	S	S	S
P	R	R	R	R	R	R	R	R
S	R	R	R	S	R	S	R	S
SXT	R	R	R	R	S	S	S	R
TE	R	R	R	S	R	R	R	S
TOB	S	R	R	S	S	S	S	S
VA	S	S	S	S	S	S	S	S

Antibiotics: AMC: amoxicillin + clavulanic acid; AMP: ampicillin; CRO: ceftriaxone; CD: clindamycin; CIP: ciprofloxacin; E: erythromycin; ENR: enrofloxacin; CN: gentamicin; IMI: imipenem; LNZ: linezolid; OX: oxacillin, P: penicillin, S: streptomycin; SXT: sulfamethoxazole + trimethoprim; TE: tetracycline; TOB: tobramycin; VA: vancomycin.

**Table 3:** Chemical composition of essential oils.

Compounds	LRI ^1^	Class ^2^	*At*	*Ca*	*Cc*	*Cm*	*Cs*	*Cz*	*Eg*	*Ls*	*Mo*	*Ob*	*Sm*
α-Pinene	939	mh	1.1	1.2	0.2		6.2	0.3	2.3	1.6		0.5	0.9
Sabinene	975	mh	26.0	0.6								0.5	
β-Pinene	979	mh					2.8	0.1	0.1			1.1	
6-Methyl-5-hepten-2-one	986	nt	0.2		1.5						1.0		
Myrcene	991	mh	0.5	3.5			16.1		0.1	0.2	0.2	0.9	1.3
α-Terpinene	1017	mh					0.2	0.3					1.3
p-Cymene	1025	mh	0.5					1.3		0.3			10.3
o-Cymene	1026	mh					0.1		8.0			0.2	
Limonene	1029	mh	31.1	92.6	1.5	0.4	2.0		4.3		0.1	0.5	3.0
β-Phellandrene	1030	mh						1.7					
1,8-Cineole	1033	om	6.1		0.2				84.2			10.7	
(*Z*)-β-Ocimene	1037	mh	0.1		0.1		1.2						
(*E*)-β-Ocimene	1050	mh	2.4				7.1				0.2	0.4	
γ-Terpinene	1060	mh	0.2				0.2						6.2
Terpinolene	1089	mh					14.2					0.1	
Linalool	1097	om	3.4	0.5	1.1			3.1		0.1	0.3	46.0	1.5
Citronellal	1158	om	11.2		0.6						8.1		
Borneol	1169	om											3.0
Isoneral	1170	om			0.8						1.2		
4-Terpineol	1177	om	0.8				0.1	0.2				0.2	1.6
Isogeranial	1185	om			1.1						1.8		
α-Terpineol	1189	om	0.6		0.2			0.6				0.8	1.4
Estragole	1196	pp										1.3	
*trans*-Isopiperitenol	1210	om	1.5										
Citronellol	1226	om	3.3		0.5						2.0		
Neral	1238	om	0.8		32.6						29.0		
*iso*-Thymol methyl ether	1244	om											5.2
Geraniol	1253	om	0.1		5.0						1.8		
Geranial	1267	om	1.4		40.1						36.5		
(*E*)-Cinnamaldehyde	1270	nt						63.2					
Bornyl acetate	1289	om										1.3	
Thymol	1290	om											7.0
Carvacrol	1299	om											45.4
α-Cubebene	1351	sh								3.3			
Eugenol	1359	pp						3.5				2.3	
α-Copaene	1377	sh	0.2					0.7		5.2	0.1	0.3	0.3
Geranyl acetate	1381	om	0.6		4.5						1.7		
β-Elemene	1391	sh				6.9						3.4	
β-Caryophyllene	1419	sh	1.5		2.4	0.4	20.8	6.2		0.5	9.0	0.4	3.5
*trans*-α-Bergamotene	1435	sh					1.9					8.0	
α-Guaiene	1440	sh								2.6		1.1	0.3
Cinnamyl acetate	1445	nt						3.5					
α-Humulene	1455	sh			0.2	0.2	6.9	1.2			0.5	1.0	
(*E*)-β-Farnesene	1457	sh					2.1					0.1	
γ-Muurolene	1480	sh								1.8			0.2
Germacrene D	1485	sh				1.5					1.5	3.5	
β-Selinene	1490	sh				0.9	1.2			3.9			0.2
α-Selinene	1494	sh				0.8	1.0			3.3			
Curzerene	1495	sh				17.5							
*trans*-β-Guaiene	1503	sh								1.1			
α-Bulnesene	1510	sh				0.8						2.2	
γ-Cadinene	1513	sh						6.2					
*trans*-γ-Cadinene	1514	sh			1.6							3.8	
*cis*-Calamenene	1540	sh								22.7			
Selina-3,7(11)-diene	1542	sh					1.8						
Flavesone	1547	nt								7.2			
Germacrene B	1561	sh				5.2	0.7						
Spathulenol	1578	os								1.2		0.2	
Caryophyllene oxide	1583	os			1.0		3.5	1.3			0.6		
Globulol	1585	os							0.1	2.8			
*iso*-Leptospermone	1621	os								7.0			
Leptospermone	1629	os								19.2			
*epi*-α-Cadinol	1640	os										3.1	
*t*-Cadinol	1643	os								1.4			
Furanoeudesma-1,3-diene	1645	os				33.7							
Lindestrene	1652	os				11.9							
*cis*-Calamene-10-o	1661	os								1.0			
Atractylone	1669	os				9.8							
cadalene	1676	sh								1.0			
Germacrone	1694	os				1.0							
(*R*,5*E*,9*E*)-8-Methoxy-3,6,10-trimethyl-4,7,8,11-tetrahydrocyclodeca[b]furan	1733	os				5.6							
Benzyl benzoate	1760	nt						2.6					
*m-*Camphorene	1960	dh					1.5						
Pentacosane	2500	nt					1.5						
^2^Class of Compounds													
Monoterpene hydrocarbons (mh)			62.1	98.5	2.6	0.4	51.4	4.4	14.8	2.1	0.5	4.3	23
Oxygenated monoterpenes (om)			34.6	0.5	88.4		0.5	3.9	84.8	0.1	85.3	59.8	65.1
Sesquiterpene hydrocarbons (sh)			1.9	0.6	4.6	36.2	38.7	14.5	0.2	45.4	11.3	27.6	4.5
Oxygenated sesquiterpenes (os)					1.2	63.2	4.4	1.4	0.1	32.6	0.7	3.8	
Diterpenes hydrocarbons (dh)							2.1						
Oxygenated diterpenes (od)					0.1		0.4						
Phenylpropanoids (pp)					0.2			3.5				3.8	
Non-terpene derivatives (nt)			1.3	0.4	2.8		1.6	71.7		7.2	1.8	0.2	
Total compounds identified			99.9	100	99.9	99.8	99.1	99.4	99.9	87.6	99.6	99.5	92.6

^1^ LRI: linear retention indices on DB-5 column. Class ^2^: Class: class of compounds as described above. At, Aloysia triphylla; Ca, Citrus aurantium; Cc, Cymbopogon citratus; Cm, Commiphora molmol; Cs, Cannabis sativa; Cz, Cinnamomum zeylanicum; Eg, Eucalyptus globulus; Ls, Leptospermum scoparium; Mo, Melissa officinalis; Ob, Ocimum basilicum; Sm, Satureja montana. Identified compounds with abundance ≤1% were not inserted in this table, but they were utilized for calculating the sums of the classes.

**Table 4:** Minimum inhibitory concentration (MIC) modal values of the tested essential oils against the eight isolates.

Isolates	*At*	*Ca*	*Cc*	*Cm*	*Cs*	*Cz*	*Eg*	*Ls*	*Mo*	*Ob*	*Sm*
1	1:32	1:32	1:256	1:64	1:16	1:1024	1:128	1:512	1:1024	1:32	1:512
2	1:32	1:32	1:256	1:64	1:16	1:1024	1:256	1:512	1:512	1:64	1:256
3	1:32	1:32	1:256	1:64	1:16	1:1024	1:256	1:1024	1:1024	1:32	1:512
4	1:32	1:32	1:256	1:64	1:16	1:1024	1:256	1:1024	1:1024	1:64	1:512
5	1:32	1:32	1:1024	1:256	1:32	1:1024	1:64	1:1024	1:1024	1:64	1:1024
6	1:64	1:64	1:1024	1:256	1:32	1:1024	1:64	1:512	1:1024	1:64	1:512
7	1:128	1:32	1:1024	1:256	1:32	1:1024	1:64	1:1024	1:1024	1:64	1:1024
8	1:64	1:32	1:1024	1:256	1:32	1:1024	1:128	1:512	1:2048	1:64	1:512

Isolates 1, 2, 3, and 4 were classified as methicillin-resistant *Staphylococcus pseudintermedius* (MRSP). Isolates 5, 6, 7, and 8 were classified as methicillin-susceptible *Staphylococcus pseudintermedius* (MSSP). *At, Aloysia triphylla; Ca, Citrus aurantium; Cc, Cymbopogon citratus; Cm, Commiphora molmol; Cs, Cannabis sativa; Cz, Cinnamomum zeylanicum; Eg, Eucalyptus globulus; Ls, Leptospermum scoparium; Mo, Melissa officinalis; Ob, Ocimum basilicum; Sm, Satureja montana*.

**Table 5:** Minimum bactericidal concentration (MBC) modal values of the tested essential oils against the eight isolates.

Isolates	*At*	*Ca*	*Cc*	*Cm*	*Cs*	*Cz*	*Eg*	*Ls*	*Mo*	*Ob*	*Sm*
1	1:16	1:16	1:256	1:32	1:8	1:1024	1:64	1:256	1:512	1:16	1:256
2	1:32	1:16	1:256	1:64	1:8	1:1024	1:256	1:256	1:512	1:32	1:128
3	1:16	1:16	1:256	1:32	1:8	1:1024	1:128	1:512	1:512	1:16	1:256
4	1:32	1:16	1:256	1:64	1:8	1:512	1:128	1:512	1:512	1:32	1:256
5	1:32	1:16	1:512	1:128	1:8	1:512	1:64	1:512	1:1024	1:32	1:512
6	1:32	1:32	1:1024	1:128	1:8	1:1024	1:64	1:256	1:512	1:32	1:256
7	1:64	1:16	1:512	1:128	1:16	1:512	1:64	1:512	1:1024	1:32	1:512
8	1:32	1:16	1:1024	1:128	1:8	1:1024	1:64	1:256	1:1024	1:32	1:256

Isolates 1, 2, 3, and 4 were classified as methicillin-resistant *Staphylococcus pseudintermedius* (MRSP). Isolates 5, 6, 7, and 8 were classified as methicillin-susceptible *Staphylococcus pseudintermedius* (MSSP). *At, Aloysia triphylla*; *Ca, Citrus aurantium*; *Cc, Cymbopogon citratus*; *Cm, Commiphora molmol*; *Cs, Cannabis sativa*; *Cz, Cinnamomum zeylanicum*; *Eg, Eucalyptus globulus*; *Ls, Leptospermum scoparium*; *Mo, Melissa officinalis*; *Ob, Ocimum basilicum; Sm, Satureja montana*.
